# Correction: Neuroendocrine modulation sustains the *C. elegans* forward motor state

**DOI:** 10.7554/eLife.26528

**Published:** 2017-03-08

**Authors:** Maria Lim, Jyothsna Chitturi, Valeriya Laskova, Jun Meng, Daniel Findeis, Anne Wiekenberg, Ben Mulcahy, Linjiao Luo, Yan Li, Yangning Lu, Wesley Hung, Yixin Qu, Chiyip Ho, Douglas Holmyard, Ni Ji, Rebecca D McWhirter, Aravinthan DT Samuel, David M Miller, Ralf Schnabel, John A Calarco, Mei Zhen

Lim MA, Chitturi J, Laskova V, Meng J, Findeis D, Wiekenberg A, Mulcahy B, Luo L, Li Y, Lu Y, Hung W, Qu Y, Ho C-Y, Holmyard D, Ji N, McWhirter R, Samuel ADT, Miller DM, Schnabel R, Calarco JA, Zhen M. 2016. Neuroendocrine modulation sustains the C. elegans forward motor state. *eLife* **5**:e19887. doi: 10.7554/eLife.19887.

Published 18, November 2016

We made a modification to a cartoon drawing of the RID neurite morphology in Figure 1A. The original sketch may be interpreted as the ventral-dorsal branch of the RID neurite loops around the pharynx. The new version prevents any incorrect interpretation of the trajectory of this neurite. It does not run in a circle around the pharynx, as might be implied by the cartoon, but rather loops back around the left side of the animal *en route* to the dorsal nerve cord. We have corrected the Figure to avoid any potential confusion. The original article did not make any statement on the trajectory. We thank our colleagues who brought our attention to this potential misinterpretation of the original sketch.

The corrected Figure 1 is shown here:

**Figure 1:**
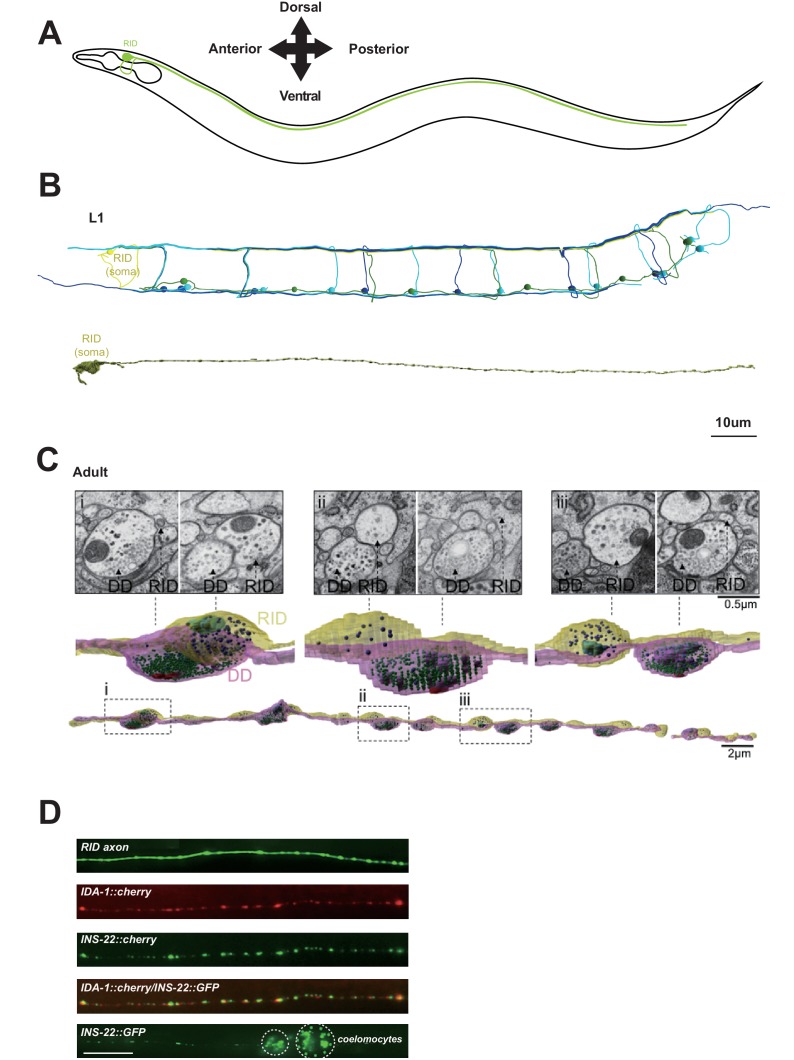

The originally published Figure 1 is also shown for reference:

**Figure 2:**
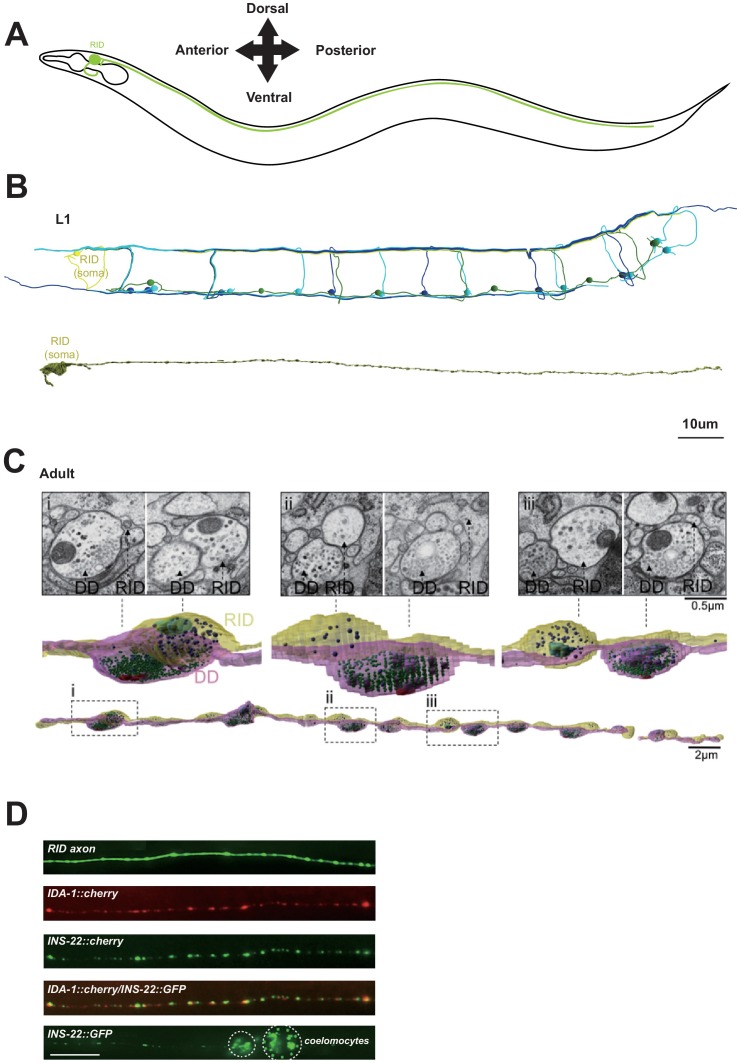

The article has been corrected accordingly.

