# Supplementary material for: Neuroendocrine modulation sustains the C. elegans forward motor state
Source: eLife. 2016 Nov 18;5:e19887. doi: 10.7554/eLife.19887 (PMC5120884; doi:10.7554/eLife.19887)
Supplement: Supplementary file 1. — DOI: http://dx.doi.org/10.7554/eLife.19887.025 [file elife-19887-supp1.docx]

**Table S1. Identification of Enriched Neuropeptide Transcripts in RID by Subtractive Transcriptome Profiling: Results for transcripts (both positive and negative for enrichment) that validated the dataset analyses pipeline**

| **Gene** | | | **Average Transcript Counts**  **(Mean+SD)** | | | **Average Transcript Counts**  **(Mean+SD)** | | **Enrichment in GFP+ Cells**  **(Fold change)** | | **P values for the Enrichment**  **(FDR-corrected; n>=3 replica)** | |
| --- | --- | --- | --- | --- | --- | --- | --- | --- | --- | --- | --- |
| **Class** | **Sequence ID (Gene Name)** | | **GFP+ cells (wt)** | | **All cells (wt)** | **GFP+ cells**  **(*unc-39 mutants*)** | **All cells**  **(*unc-39 mutants*)** | ***wild type*** | ***unc-39 mutants*** | ***wild-type*** | ***unc-39 mutants*** |
| **Similarly enriched in wild-type (WT) and *unc-39* GFP+ cells – transcripts known to be expressed by shared GFP+ cells (positive controls)** | | | | | | | | | | | |
| ***Previously reported*** | | | | | | | | | | | |
| Pair homeobox transcription factor | | W03A3.1 (*ceh-10)* | 121594.1 + 561.1 | | 8.3 + 5.8 | 3132.3 + 2037.8 | 7.0 + 4.8 | 191.1 | 445.1 | 9.74E-22 | 7.28E-08 |
| LIM homeobox transcription factor | | C40H5.5a (*ttx-3)* | 1028.7 + 402.9 | | 14.4 + 6.8 | 2323.0 + 1331.1 | 17.3 + 16.1 | 71.4 | 134.6 | 3.21E-13 | 1.45E-07 |
| Glutamate-gated chloride channel subunit | | ZC317.3 (*glc-3)* | 1139.2 + 192.4 | | 63.9 + 37.0 | 2156.2 + 1403.8 | 52.0 + 40.9 | 17.8 | 41.4 | 4.56E-16 | 3.57E-04 |
| Serotonin/  octopamine receptor | | C02D4.2a (*ser-2)* | 4806.8 + 1238.0 | | 362.9 + 83.6 | 7086.3 + 3171.4 | 275.5 + 92.6 | 13.3 | 25.7 | 1.68E-14 | 1.38E-07 |
| Kallmann syndrome-associated membrane protein | | K03D10.1 (*kal-1)* | 1041.1 + 40.2 | | 380.4 + 118.1 | 2136.0 + 1192.0 | 411.3 + 92.3 | 2.7 | 5.2 | 0.02 | 0.02 |
| ***Secreted*** | | | | | | | | | | | |
| Secreted protein | | C36B7.7  (*hen-1)* | 629.7 + 239.4 | | 16.1 + 5.0 | 1218.2 + 822.9 | 22.6 + 13.1 | 39.1 | 54.0 | 3.23E-11 | 4.22E-04 |
| ***Related to neurotransmitters or neuromodulators*** | | | | | | | | | | | |
| Monoamine transporter | | W01C8.6  (*cat-1)* | 733.4 + 121.4 | | 87.3 + 31.5 | 847.5 + 272.7 | 60.1 + 35.6 | 8.4 | 14.1 | 1.48E-08 | 1.76E-09 |
| Acetylcholine transporter | | ZC416.8  (*unc-17)* | 940.4+ 104.2 | | 200.0 + 73.1 | 1254.3 + 516.3 | 265.0 + 84.9 | 4.7 | 4.7 | 2.18E-05 | 1.26E-03 |
| Choline acetyltransferase | | ZC416.8  (*cha-1)* | 422.1 + 92.2 | | 141.3 + 31.1 | 565.7 + 243.0 | 141.7 + 80.3 | 3.0 | 4.0 | 1.23E-02 | 4.72E-02 |
| NLP-family peptide | | F48B9.4  (*pdf-2)* | 11535.6 + 3721.3 | | 89.4 + 21.0 | 18096.1 + 9039.8 | 76.5 + 29.0 | 129.0 | 236.7 | 3.33E-23 | 2.21E-11 |
| NLP-family peptide | | T07E3.6a (*pdf-1)* | 263951.0 + 30655.9 | | 6652.6 + 384.5 | 318710.4 + 146449.1 | 8937.4 + 1669.5 | 39.7 | 35.7 | 4.67E-26 | 1.16E-07 |
| FLP-family peptide | | T06C10.4  (*flp-10)* | 263951.0 + 30655.9 | | 6652.6 + 384.5 | 2894.0 + 1488.8 | 61.7 + 38.2 | 31.8 | 46.9 | 2.61E-21 | 1.24E-06 |
| Caenacin-family peptide | | R09B5.13 (*cnc-11)* | 306.8 + 190.9 | | 11.7 + 3.64 | 424.1 + 216.3 | 20.1 + 18.5 | 26.3 | 21.1 | 5.15E-03 | 6.42E-05 |
| NLP-family  peptide | | CC4.2  (*nlp-15)* | 27310.8 + 2827.4 | | 1826.4 + 237.8 | 63296.2 + 40301.7 | 1886.8 + 451.8 | 15.0 | 33.6 | 4.04E-15 | 5.26E-04 |
| NLP-family  peptide | | C04H5.8a (*nlp-41)* | 378.4 + 33.1 | | 26.2 + 14.9 | 587.9 + 320.6 | 17.8 + 2.6 | 14.5 | 33.0 | 1.36E-11 | 2.52E-05 |
| NLP-family  peptide | | D1009.4a (*nlp-14)* | 4574.2 + 788.3 | | 329.6 + 64.3 | 4781.8 + 1977.6 | 362.0 + 89.6 | 13.9 | 13.2 | 7.35E-14 | 2.84E-06 |
| FLP-family  peptide | | C26F1.10  (*flp-21)* | 295.0 + 37.0 | | 28.0 + 10.5 | 422.5 + 172.9 | 65.4 + 27.3 | 10.5 | 6.5 | 4.15E-08 | 4.69E-04 |
| Caenacin-family peptide | | VK10D6R.1 (*cnc-10)* | 171.3 + 36.6 | | 20.1 + 3.4 | 604.3 + 446.5 | 28.8 + 13.4 | 8.5 | 21.0 | 1.32E-05 | 0.01 |
| Insulin-family  pepetide | | F13B12.5 (*ins-1)* | 491.9 + 93.3 | | 70.1 + 13.8 | 454.5 + 180.8 | 37.9 + 2.4 | 7.0 | 12.0 | 1.29E-06 | 1.48E-06 |
| FLP-family  peptide | | Y48D7A.2 (*flp-18)* | 454.5 + 180.8 | | 37.8 + 2.4 | 2604.1 + 1174.6 | 282.4 + 74.8 | 6.2 | 9.2 | 5.84E-07 | 1.79E-04 |
| **Not enriched in wild-type and *unc-39* GFP cells - transcripts expected to be absent from all GFP cells (negative controls)** | | | | | | | | | | | |
| ***Muscle-specific*** | | | | | | | | | | | |
| Myosin  heavy chain | T18D3.4 (*myo-2)* | | 4457.8 + 2753.5 | 3304.0 + 1739.4 | | 2801.5 + 2571.1 | 3343.8 + 1804.0 | 1.4 | 0.8 | 1 | 1 |
| Myosin  heavy chain | K12F2.1  (*myo-3)* | | 1423.6 + 3783.5 | 3783.5 + 1535.8 | | 943.8 + 3783.5 | 1535.8 + 1423.6 | 0.4 | 0.6 | 0.10 | 0.13 |
| ***Other neurotransmitters*** | | | | | | | | | | | |
| Vesicular glutamate transporter | ZK512.6a (*eat-4)* | | 175.6 + 74.5 | 209.3 + 60.4 | | 151.8 + 61.9 | 160.8 + 104.6 | 0.8 | 0.9 | 1 | 1 |
| Glutamic acid decarboxylase | Y37D8A.23a (*unc-25)* | | 4499.3 + 700.6 | 6901.5 + 2031.0 | | 4499.3 + 701.0 | 6901.5 + 2031.0 | 0.6 | 0.6 | 1 | 0.74 |
| **Candidates for RID-enriched transcripts: preferentially enriched in wild-type GFP cells** | | | | | | | | | | | |
| ***Non-peptidergic (transcripts known to be present in RID; positive controls)*** | | | | | | | | | | | |
| Secreted protein with Ig domains | Y48A6A.1 (*zig-5)* | | 2604.1 + 1174.6 | 282.4 + 74.8 | | 560.0 + 545.1 | 93.6 + 32.1 | 15.8 | 6.0 | 4.85E-14 | 0.35 |
| LIM homeobox transcription factor | ZC64.4  (*lim-4)* | | 1558.5 + 637.5 | 137.3 + 13.9 | | 783.9 + 697.6 | 95.3 + 29.5 | 11.4 | 8.2 | 1.01E-05 | 0.17 |
| ***Peptidergic (RID enriched)*** | | | | | | | | | | | |
| Insulin-family  peptide | F56F3.6  (*ins-17)* | | 265.6 + 37.8 | 34.4 + 10.5 | | 101.7 + 68.7 | 56.0 + 17.8 | 7.7 | 1.8 | 2.37E-06 | 0.72 |
| FLP-family peptide | Y37D8A.15 (*flp-14)* | | 19547.2 + 586.8 | 2917.5 + 368.0 | | 8562.1 + 6291.9 | 3427.3 + 1069.2 | 6.7 | 2.5 | 7.55E-08 | 0.53 |
| NLP-family peptide | B0213.17  (*nlp-34)* | | 67.0 + 23.2 | 9.8 + 6.5 | | 37.8 + 35.3 | 13.3 + 6.5 | 6.8 | 2.8 | 0.01 | 0.67 |
